# Spatio-Temporal Patterns and Ecological Implications of Microplastic Contamination in Benthic Fish from the Tongkou River, China

**DOI:** 10.3390/ani16203177

**Published:** 2026-10-10

**Authors:** Jiayun Wu, Qingqing Liao, Yingying Ren, Linhong Zhang, Qishen Tan, Yijun Li, Qixiang Li, Yunkun Li, Xiaogang Du, Shiyong Yang

**Affiliations:** 1 College of Life Sciences, Sichuan Agricultural University, Ya’an 625000, China; wujy@sicau.edu.cn (J.W.);; 2 Aquatic Health and Intelligent Aquaculture Key Laboratory of Sichuan Province, College of Fisheries, Sichuan Agricultural University, Chengdu 611130, China

**Keywords:** synthetic polymers, aquatic environment, bioaccumulation, Raman spectroscopy

## Abstract

Microplastic pollution in rivers is a critical environmental threat that harms aquatic life. To understand the abundance, physical characteristics, and polymer types of microplastics, this study examined the intestinal tract of four fish groups (three individual species: *Saurogobio dabryi*, *Squalidus argentatus*, and *Hemibarbus labeo*; and a pooled group of four loach species: *Paramisgurnus dabryanus*, *Triplophysa bleekeri*, *Oreias dabryi*, and *Paracobitis variegatus*) collected from the Tongkou River in October 2023 and November 2024 using tissue digestion methods across different river sections. The results revealed that every single fish contained microplastics, with contamination levels significantly higher downstream than upstream. Over the study period, total microplastic counts dropped by 32 percent from 2023 to 2024. Most of the collected pellets were thin threads and small fragments measuring under one millimeter, composed mainly of common plastics and cellulose. These findings demonstrate that freshwater fish are widely exposed to small plastic fibers, showing distinct geographic and yearly trends. Ultimately, this research provides essential baseline data to help authorities develop effective strategies to reduce plastic pollution in freshwater ecosystems and protect aquatic wildlife.

## Simple Summary

Microplastic pollution in rivers poses an increasing threat to freshwater fish and the ecosystems they rely on. To investigate this issue, we examined the intestinal tracts of eight fish species collected from the Tongkou River in October 2023 and November 2024. We aimed to characterize the abundance, morphology, and polymer composition of ingested microplastics. Microplastics were detected in every fish species examined, with contamination levels significantly higher at downstream sites than at upstream sites. Notably, total microplastic counts decreased by 32% from 2023 to 2024. The majority of particles identified were fibers and small fragments measuring under one millimeter, primarily composed of common plastics and cellulose-based materials. These results confirm widespread exposure of freshwater fish to microplastic pollution, with pronounced spatial and temporal variation. This study provides critical baseline data to support evidence-based strategies for reducing plastic pollution and safeguarding aquatic life in freshwater ecosystems.

## 1. Introduction

Microplastics (MPs), defined as small plastic pollutants with a diameter of less than 5 mm, have emerged as significant environmental contaminants [1]. Their persistence, potential for bioaccumulation, and toxic effects on ecosystems have garnered widespread attention in global environmental and health research. Predictions suggest that by 2050, the total amount of plastic waste entering the natural environment will reach 12 billion tons [2]. In recent years, researchers have identified microplastics in various regions worldwide, including lakes, rivers, oceans, continents, and polar areas [3]. Accumulated in the natural environment, plastic waste slowly degrades into tiny plastic pollutants under the influence of natural factors such as light, oxidation, and weathering [4]. Microplastics pose a severe ecological threat through composite pollution, as they can alter the bioavailability of co-existing pollutants and the stress responses they induce in organisms [5]. Beyond acting as direct stressors to organisms, microplastics can also impact higher trophic level organisms through the food chain [6]. As the largest consumers in nature, humans have been confirmed to ingest microplastics via the food chain, posing a potential threat to life and health. Furthermore, due to their small size and resemblance to food, microplastics are easily ingested by aquatic organisms or accumulated through pathways such as respiration and dermal adsorption [7]. This results in harm through multiple pathways, including the MPs themselves, their additives, and the pollutants they carry, leading to consequences such as cell death, altered gene expression, and intestinal damage, ultimately resulting in mortality [8,9].

The Yangtze River, the longest river in China and the third longest in the world, spans approximately 6380 km and has a basin area of 1.8 million square kilometers [10]. The Jialing River is one of the primary tributaries of the Yangtze River [11]. As the largest tributary on the right bank of the Jialing River, the Fujiang River has a total length of 720 km and a basin area of 36,400 square kilometers [12]. The Tongkou River is an important tributary of the Fujiang River system within the Yangtze River basin, characterized by a mountainous watershed with significant anthropogenic and natural influences [13]. It flows through Qushan and Tongkou before joining the Fujiang River at Qinglian Town, Jiangyou City. Beichuan Qiang Autonomous County, administered by Mianyang City, is the only Qiang autonomous county in China, with a total population of approximately 229,000 [14]. Regarding microplastic contamination, the Yangtze River Basin exhibits significant spatial heterogeneity, with reported abundances ranging from 0 to 44,080 items per cubic meter [15]. In the Min River, a tributary of the Yangtze River, water samples from the Chengdu reach showed microplastic concentrations of 6.11–44.08 n/L, with sediment loads reaching 573.84–2878.97 n/kg [16]. The Tongkou River Basin currently experiences compounded anthropogenic pressures from industrial, agricultural, and domestic pollution sources. Given the widespread accumulation and transport of emerging pollutants like microplastics across China’s freshwater basins (Zhao et al., 2022), comprehensive scientific investigation into watershed-level contamination and its ecological impacts is urgently warranted [17].

The Tongkou River Basin supports a diverse ichthyofauna, with recent surveys documenting 28 fish species classified into 22 genera, seven families, and three orders, demonstrating increased biodiversity compared to historical records [18]. The fish community is characterized by a dominance of omnivorous-benthic species, featuring key representatives such as *Squalidus argentatus* (Sauvage & Dabry de Thiersant, 1874), *Hemibarbus labeo* (Pallas, 1776), and *Schizothorax davidi* (Sauvage, 1880). Fish can accumulate microplastics from their aquatic environment over extended periods through active ingestion or passive filter-feeding, often leading to observable physiological responses [19]. Consequently, intestinal microplastic analysis in fish has become a widely adopted method for assessing aquatic microplastic pollution. Given the pervasive presence of microplastics in aquatic environments, investigating their spatio-temporal distribution in aquatic organisms is critical for assessing environmental contamination and formulating management strategies. Therefore, this study investigated the spatial and temporal distribution of microplastics in the intestinal contents of four fish categories comprising seven benthic species (including three individual species, namely *Saurogobio dabryi* (Bleeker, 1871), *Squalidus argentatus* (Sauvage & Dabry de Thiersant, 1874), and *Hemibarbus labeo* (Pallas, 1776), along with a pooled group of four loach species: *Paramisgurnus dabryanus* (Sauvage, 1878), *Triplophysa bleekeri* (Sauvage & Dabry de Thiersant, 1874), *Oreias dabryi* (Sauvage, 1874), and *Paracobitis variegatus* (Dabry de Thiersant, 1872)) across the Tongkou River Basin. Despite growing concerns regarding microplastic pollution in the Yangtze River, significant knowledge gaps remain regarding how spatial heterogeneity and fish feeding habits influence microplastic accumulation in its upstream tributaries, such as the Tongkou River. We hypothesized that microplastic abundance and characteristics in fish show significant spatial gradients linked to anthropogenic activity levels, and that benthic-feeding species accumulate higher loads due to sediment–water interactions. This study therefore aims to characterize microplastic pollution in this region and provide fundamental data for assessing microplastic pollution in the broader Yangtze River Basin.

## 2. Materials and Methods

**Figure 1:**
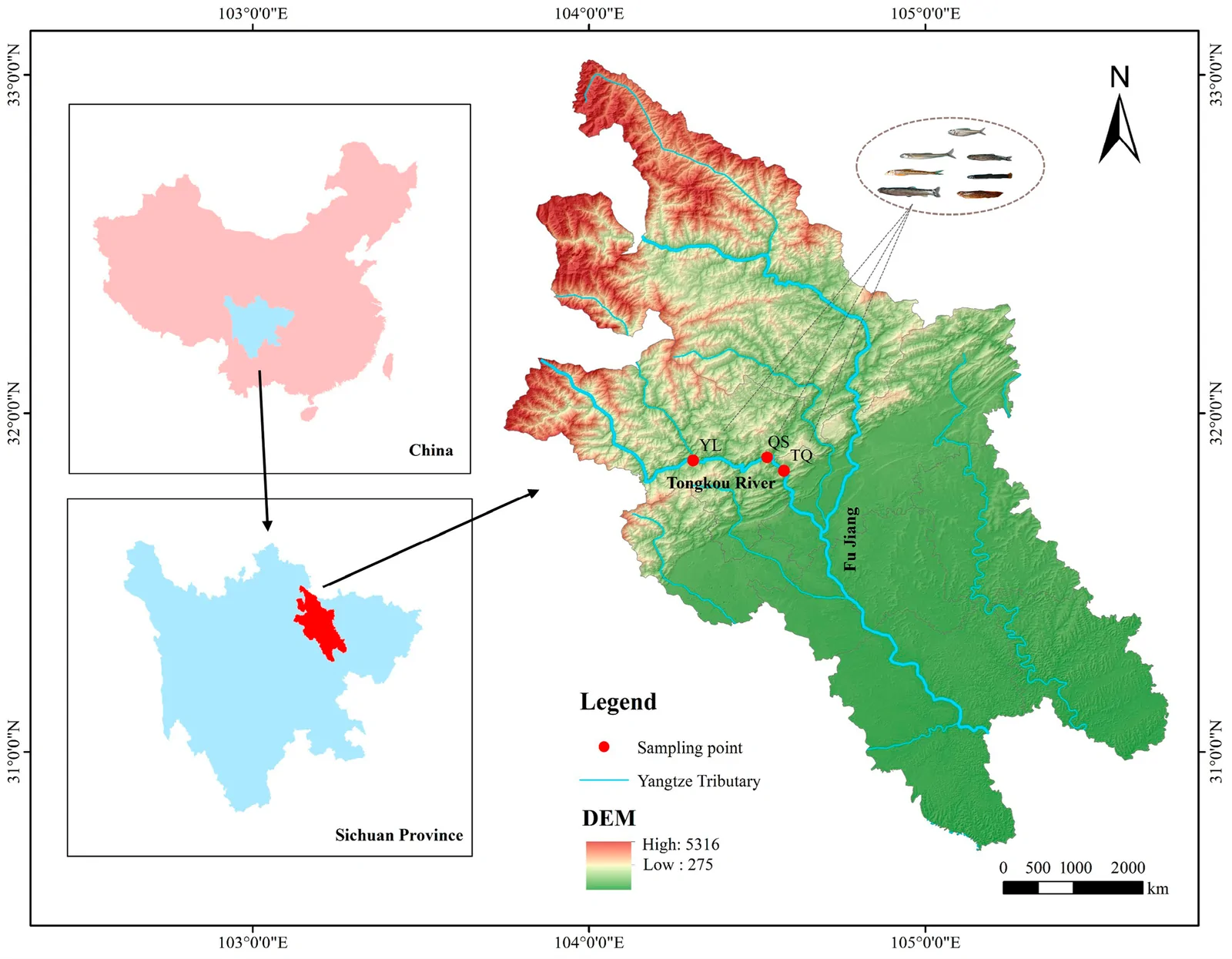
Study area and sampling points of the Tongkou River Basin, China (**YL**): Yuli Town, upper reach; (**QS**): Qushan Town, middle reach; (**TQ**): Tongquan Town, lower reach.

Fish sampling was conducted in the autumn of 2023 and 2024 using cage nets and gill nets at various collection sites (Figure 1). All collected fish specimens were preserved in anhydrous ethanol and transported to the Zoology Laboratory of Sichuan Agricultural University. A total of 152 individuals were selected for the experiment, including 31 *S. dabryi*, 27 *S. argentatus*, 34 *H. labeo*, and 60 loach specimens (12 *P. dabryanus*, 18 *T. bleekeri*, 16 *O. dabryi*, and 14 *P. variegatus*) collected from the upper Yuli Town (YL), middle Qushan Town (QS), and lower Tongquan Town (TQ) reaches of the Tongkou River (Figure 1). The body length of these specimens ranged from 5.2 to 20 cm, and the body weight ranged from 1.3 to 18 g. Prior to the experiment, all utensils were rinsed three times with filtered deionized water, and all solutions were pre-filtered through glass fiber filters (GF/F, 0.7 μm pore size, 47 mm diameter). To monitor and minimize potential background contamination from the laboratory environment, strict quality control (QC) measures were implemented. Procedural blanks (consisting of all reagents and clean glassware processed without fish samples) and air blanks (open Petri dishes containing ultrapure water placed adjacent to the working area during sample dissection and filtration) were conducted in parallel. Subsequent analysis revealed that background contamination was negligible. Intestinal tissues were dissected and digested with 10% potassium hydroxide at room temperature for 24 h. A saturated sodium chloride solution was added, and the mixture was allowed to stand for 24 h to facilitate the flotation of microplastics. The samples were then vacuum-filtered and rinsed with distilled water. Microplastics were preliminarily characterized for morphology, color, and quantity using a stereomicroscope (Olympus CX23, Olympus, Tokyo, Japan) and ImageJ software (version 1.8.0) and the maximum Feret’s diameter was recorded for each particle. Data are the number of microplastic fragments per fish, with each fish treated as an independent experimental unit. Normality of residuals was checked with the Shapiro–Wilk test and homogeneity of variance with Levene’s test. Differences among sampling sites and years, and their interaction, were tested using a two-way ANOVA with site and year as fixed factors. When the interaction was significant, simple effects analysis was performed, followed by Tukey’s HSD post hoc test. Data visualization was conducted using ArcGIS (version 10.2), GraphPad Prism (version 9.0), and Origin (version 2021) software. All analytical instruments, including the Raman spectrometer (ThermoFisher scientific DXR2xi‌, ThermoFisher scientific, Waltham, MA, USA), were regularly calibrated to ensure data accuracy. During spectroscopic analysis, pellets were selected based on their physical integrity and surface cleanliness to ensure the acquisition of high-quality spectra with minimal matrix interference or fluorescence. Collected microplastics were uniformly analyzed using Raman spectroscopy with a 633 nm excitation wavelength (10 mW power output). Each spectrum was acquired with an exposure time of 10 s and three accumulations to ensure a signal-to-noise ratio greater than 50:1. The acquired Raman spectra were baseline-corrected and compared against established standard polymer spectral libraries. A spectrum was preliminarily identified as a specific polymer only when the spectral similarity match exceeded 80%. This study strictly adhered to relevant laws and regulations, with all necessary special fishing permits obtained outside the fishing moratorium period. The entire investigation process was conducted under the full supervision of local fisheries authorities to ensure compliance with regulatory requirements.

## 3. Results

### 3.1. Spatiotemporal Distribution and Abundance Characteristics of Microplastics in Tongkou River Basin

**Figure 2:**
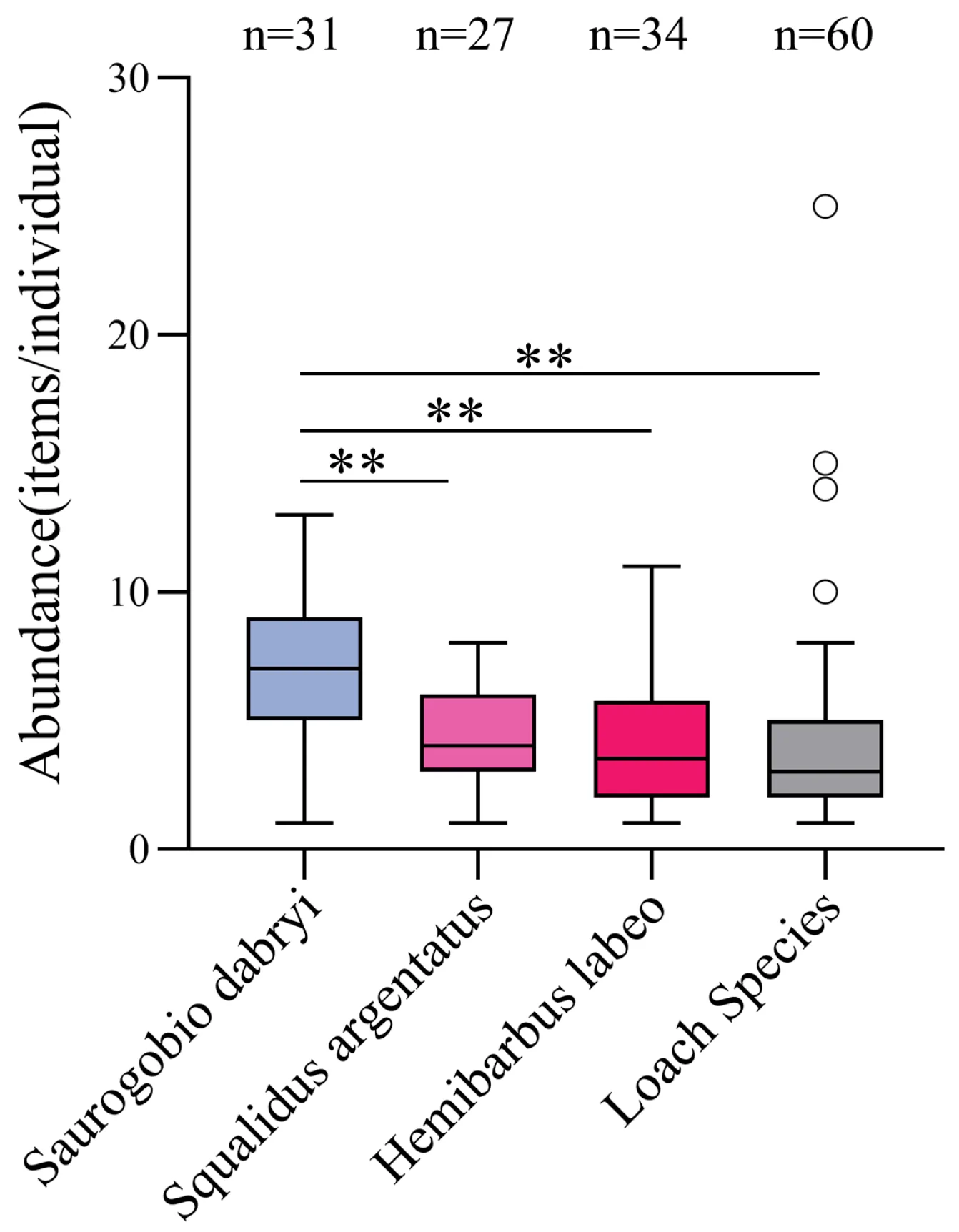
Box diagram of microplastic abundance in different benthic fishes in the Tongkou River Basin. ** indicates a statistically significant difference between the compared groups (*p* < 0.05). ° denotes outlier data points exceeding the normal range.

**Figure 3:**
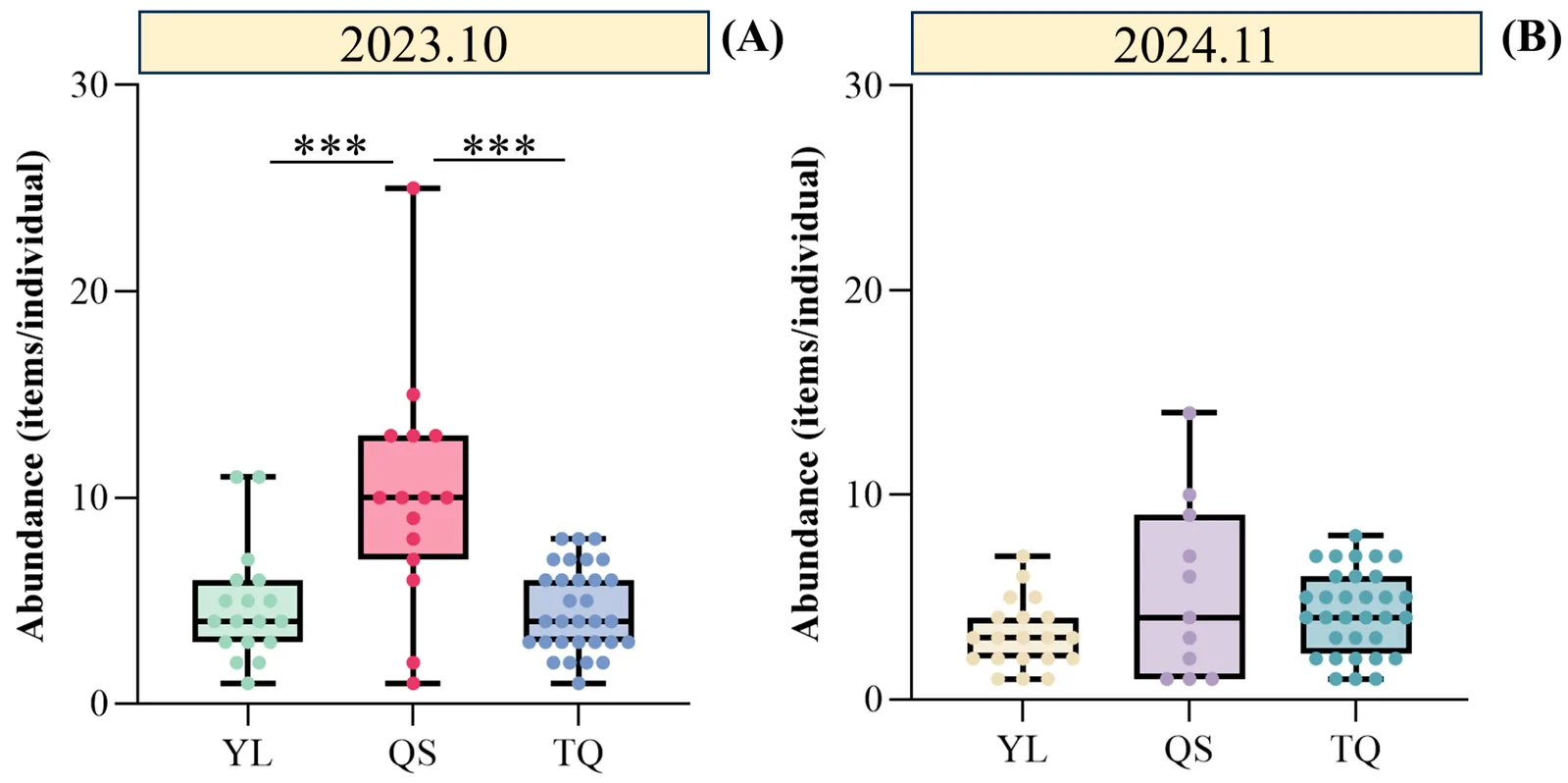
Abundance distribution of microplastics in the upper, middle and lower reaches of the Tongkou River in autumn of 2023 and 2024. (**A**) represents the microplastics distribution in 2023. (**B**) represents the microplastics distribution in 2024. *** *p* < 0.001. (**YL**): Yuli Town, upper reach; (**QS**): Qushan Town, middle reach; (**TQ**): Tongquan Town, lower reach.

Microplastic abundance across the four fish groups ranged from 1 to 25 items per individual, with a significant overall difference among the four groups (one-way ANOVA: *p* = 0.0009; Figure 2). Spatial analysis showed significantly higher concentrations in the lower reaches (TQ) compared to the upper (YL) and middle reaches (QS) (*p* less than 0.05). Furthermore, two-way ANOVA revealed a significant interaction between year and sampling site (F equals 7.03, *p* equals 0.0014) and a significant main effect of the sampling site (F equals 3.51, *p* equals 0.034), whereas the main effect of the year was marginally non-significant (F equals 3.68, *p* equals 0.058). Simple effect analysis indicated that in 2023, microplastic fragment abundance at QS was significantly higher than at YL and TQ (*p* less than 0.05), with no significant differences among sites in 2024 (Figure 3). Additionally, significant interannual variations were observed at QS and TQ (*p* less than 0.05) but not at YL, demonstrating that the spatial distribution pattern of microplastics exhibited notable interannual fluctuations. Temporally, overall contamination declined, with mean abundances decreasing by 32.1% from 2023 (total 380 items; 4.87 particles/individual) to 2024 (total 258 items; 3.49 particles/individual).

### 3.2. Morphological Characterization of Microplastics in Benthic Fish Intestines from the Tongkou River Basin

**Figure 4:**
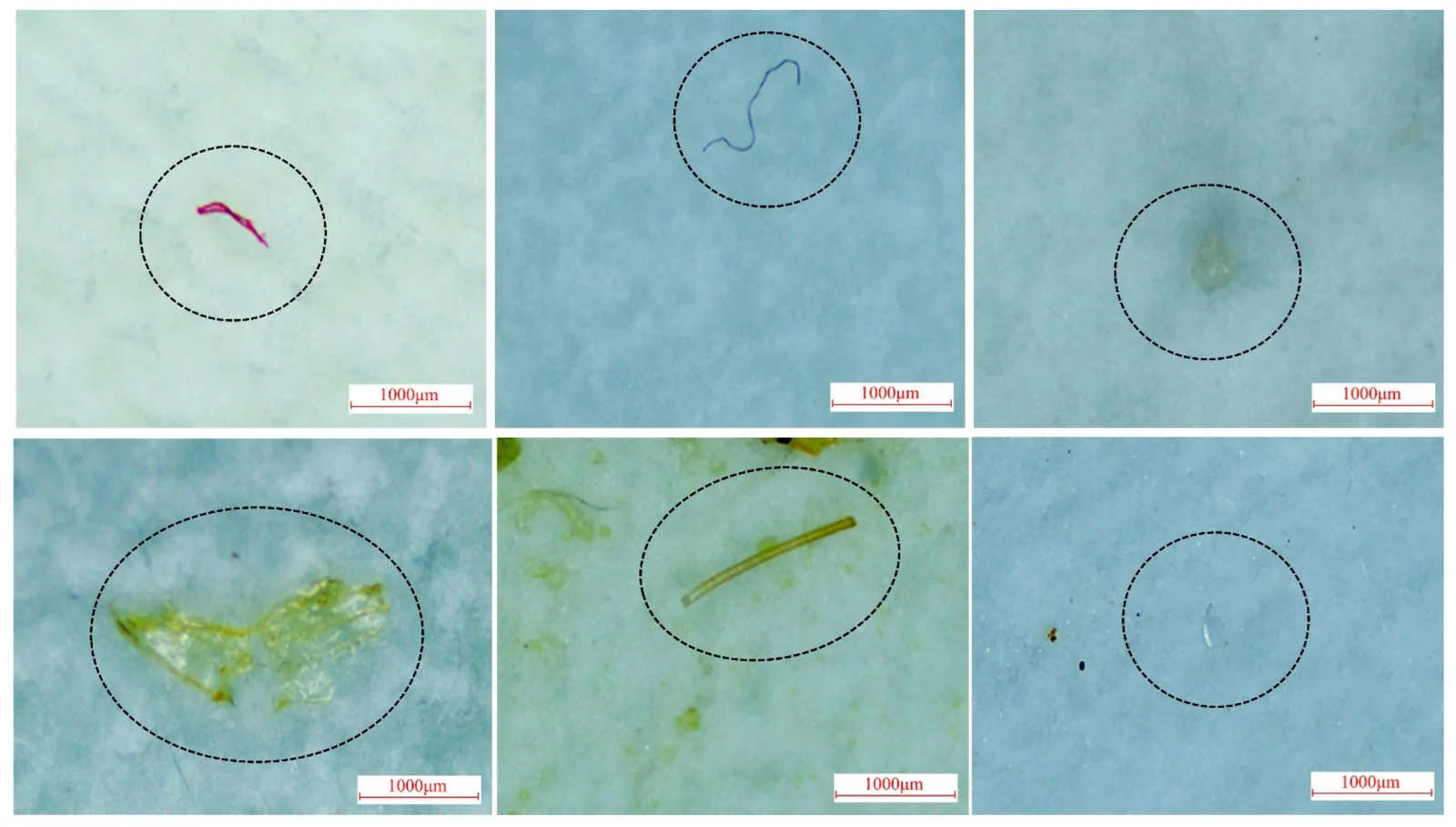
Microscopic images of typical microplastics in four fish groups in the Tongkou River Basin, China. The dashed circle marks the area containing microplastics of different morphologies.

**Figure 5:**
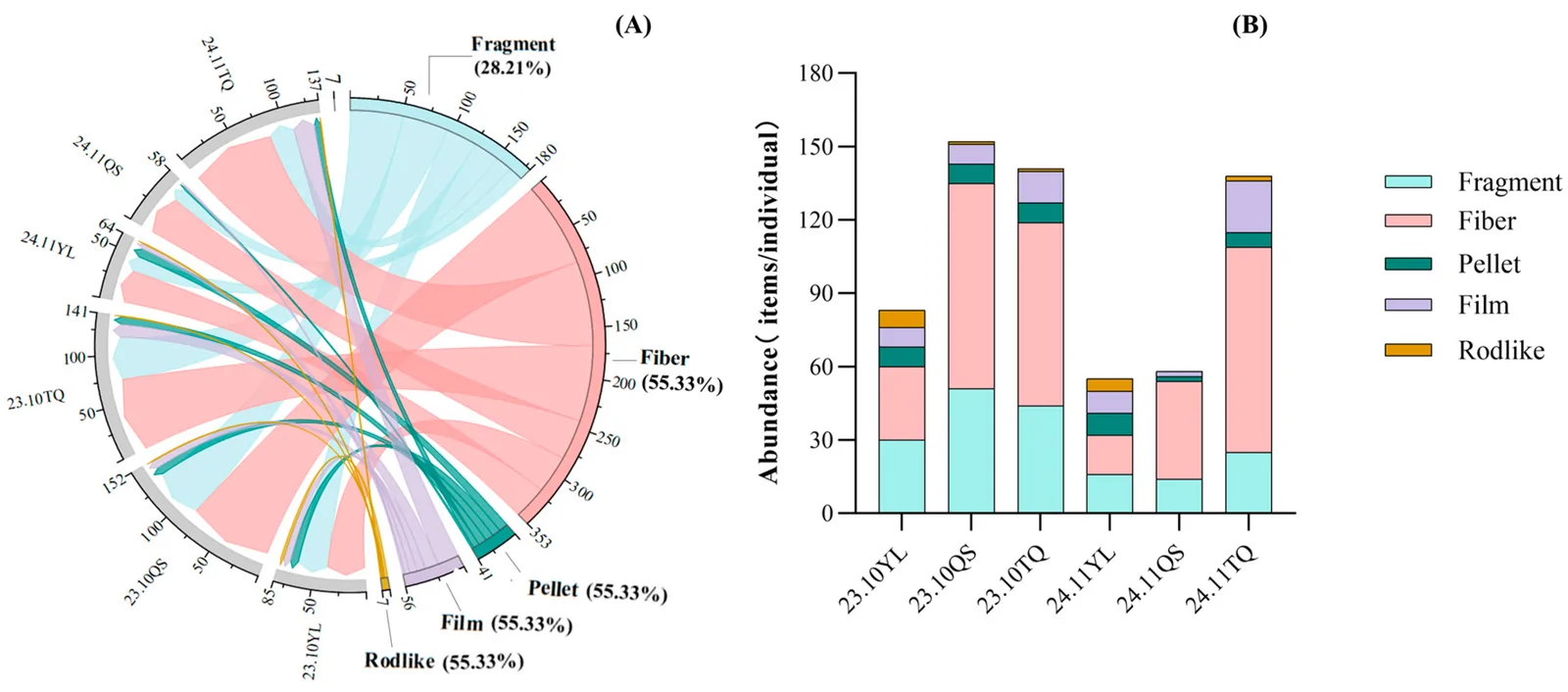
The morphological distribution of MPS in the intestinal contents of four fish groups in the Tongkou River. (**A**) Number of MPS found in the form of fragments, fibers, granules, films, and rod-like pellets. (**B**) The relative abundance of different forms of MPS. (**YL**): Yuli Town, upper reach; (**QS**): Qushan Town, middle reach; (**TQ**): Tongquan Town, lower reach.

Analysis of the isolated microplastics revealed five distinct morphological categories: fragments, fibers, pellets, films, and rodlike pellets (Figure 4). Fibrous microplastics constituted the predominant form across both spatial (middle/lower reaches) and temporal (2023–2024) dimensions, accounting for 55.33% of the total pellets. The remaining compositions included fragments (28.21%), films (8.78%), pellets (6.43%), and rodlike pellets (1.25%) (Figure 5A). A significant decline in microplastic abundance was observed between 2023 and 2024. Fibers remained the most abundant type, decreasing from 196 to 157 pellets. Similarly, fragments decreased from 125 to 55 pellets, films remained unchanged at 28 pellets, and pellets decreased from 24 to 17 pellets (Figure 5B). Rodlike pellets, which were exclusively detected in loaches (*P. dabryanus*, *T. bleekeri*, *O. dabryi* and *P. variegatus*), were rare, with five pellets identified in 2023 and two pellets in 2024. Downstream sites (TQ) exhibited the highest microplastic loads, with fibers dominating (353 pellets), followed by fragments (181), films (156), pellets (41), and rodlike pellets (7). This spatial gradient aligns with patterns observed in other riverine systems, where downstream accumulation is driven by hydrological transport.

### 3.3. Size Distribution of Microplastics in Four Benthic Fish Species from the Tongkou River Basin

**Figure 6:**
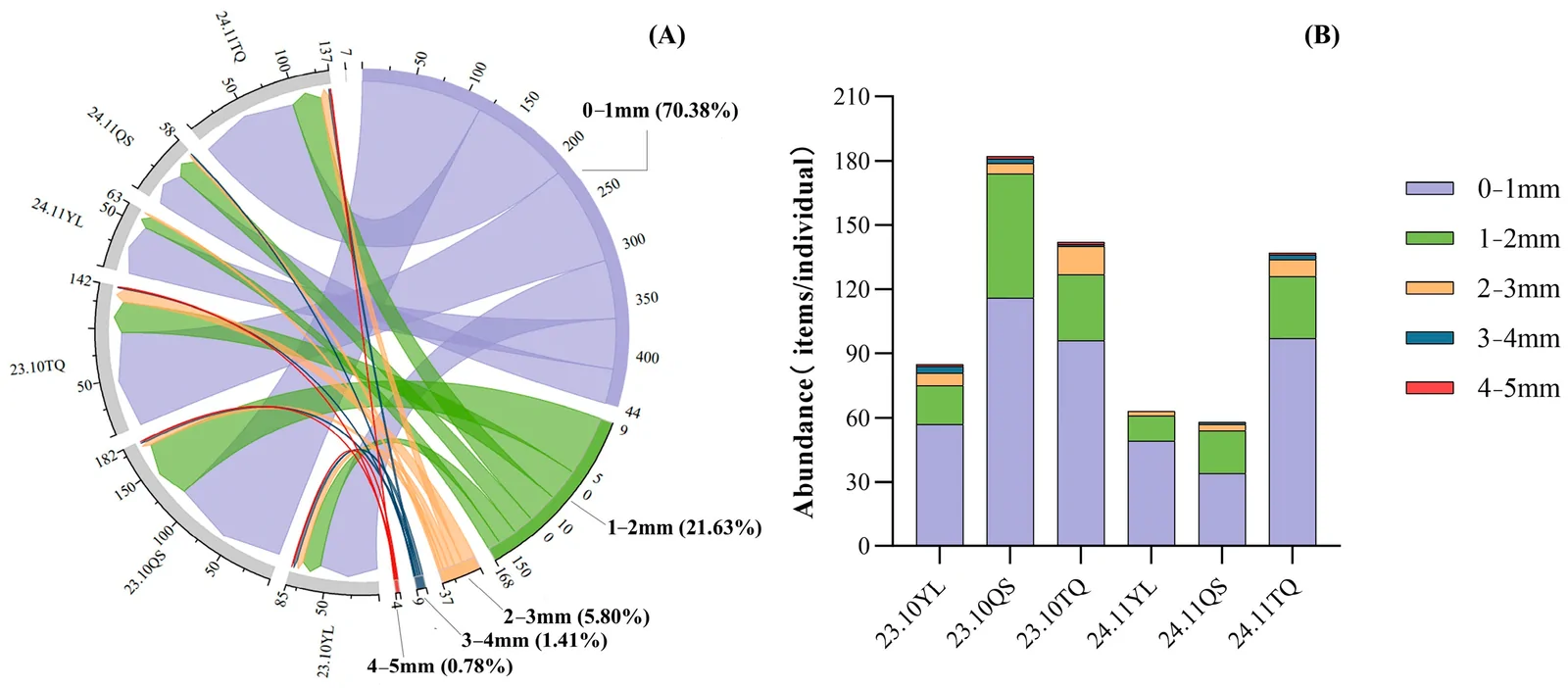
The particle size distribution of MPS in the intestinal contents of fish. (**A**) The number of MPS in different particle size ranges. (**B**) The relative abundance of MPS with different particle sizes in fish. (**YL**): Yuli Town, upper reach; (**QS**): Qushan Town, middle reach; (**TQ**): Tongquan Town, lower reach.

Microplastics isolated from the intestinal tracts of four fish species collected in the middle and lower reaches of the Tongkou River in October 2023 and November 2024 exhibited particle sizes ranging from 0.03 to 3.8 mm. The pellets were categorized into five size classes: 0–1 mm, 1–2 mm, 2–3 mm, 3–4 mm, and 4–5 mm (Figure 6A). The 0–1 mm fraction dominated, comprising 70.38% of the total pellets, which is consistent with global freshwater microplastic studies where pellets measuring ≤2 mm typically prevail. Abundance decreased with increasing size: 1–2 mm (21.63%), 2–3 mm (5.8%), 3–4 mm (1.4%), and 4–5 mm (0.78%) (Figure 6A). The total number of 0–1 mm pellets declined from 269 in 2023 to 180 in 2024, paralleling reductions in larger fractions (e.g., the 4–5 mm pellet dropped from 4 to 1) (Figure 6B). This trend aligns with observations of flood-induced microplastic redistribution in river systems. The downstream site TQ exhibited the highest load of 0–1 mm pellets (193), followed by QS (150) and YL (106), reflecting potential accumulation effects in the lower reaches. The 4–5 mm fraction was minimal, comprising only three pellets in total, suggesting limited transport of larger pellets or fragmentation during fluvial transport.

### 3.4. Polymer Composition Analysis of Microplastics

**Figure 7:**
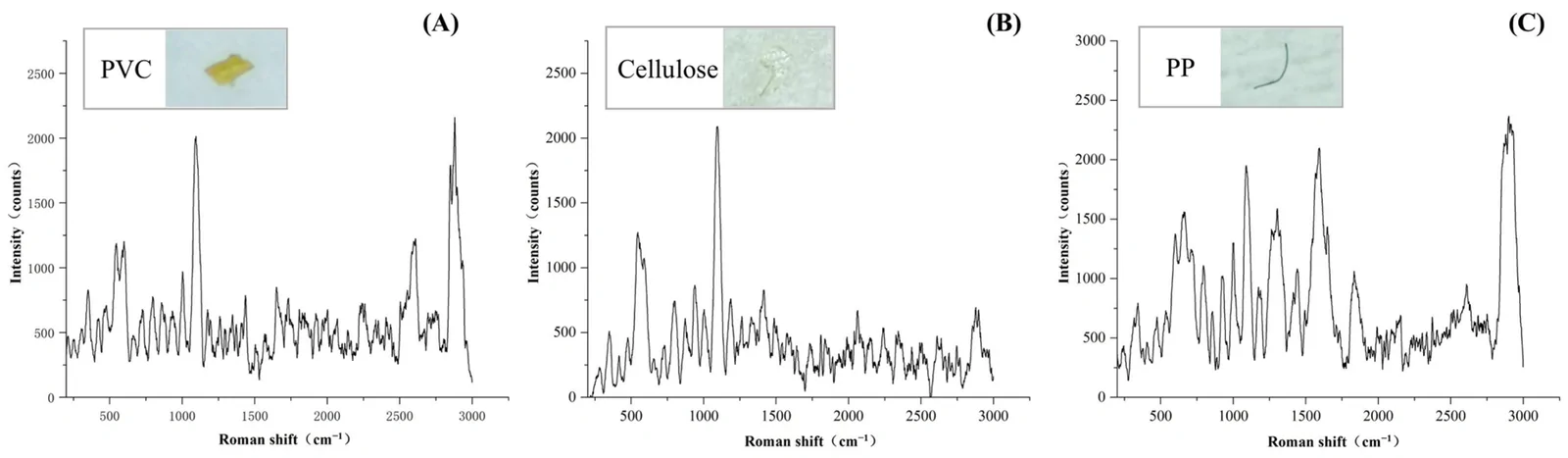
Raman spectra of microplastics in intestinal contents of fish in the Tongkou River Basin. (**A**) Raman spectra of Polyvinyl Ethyl Ether (PVE). (**B**) Raman spectra of cellulose. (**C**) Raman spectra of Polypropylene (PP).

Based on morphological characteristics, a total of representative particles (including 17 successfully verified samples) were selected for spectral analysis. Spectral identification of each tested particle revealed three distinct polymer types. The predominant component was polyvinyl chloride (PVC), which accounted for 53% of the tested subset. The Raman spectrum (Figure 7) exhibited characteristic peaks at 600 cm^−1^ and 700 cm^−1^, corresponding to C–Cl stretching vibrations, along with a prominent peak at 1000 cm^−1^ indicative of bending vibrations. Furthermore, a C–H stretching vibration peak, common to all organic compounds, was noted at 2900 cm^−1^. Cellulose was identified as a secondary component, constituting 35% of the subset. Its diagnostic spectral bands revealed a strong peak at 1096 cm^−1^, associated with C–O–C glycosidic bond vibrations, possibly originating from environmentally friendly material films. Polypropylene (PP) represented the smallest proportion at 12%, with its spectrum displaying strong C–H stretching vibration peaks in the 2800–3000 cm^−1^ range, while the region between 500–1500 cm^−1^ was dominated by C–C skeletal vibration peaks. While these proportions reflect the specific composition of the tested subset rather than every individual particle across all samples, they provide valuable insights into the primary polymer types present. Due to the limited subset analyzed, these polymer proportions reflect the compositional characteristics of the representative particles rather than the entire bulk dataset.

## 4. Discussions

As the world’s third-largest river basin, the Yangtze River faces significant ecological challenges in China. In response, China initiated the Yangtze River protection policy in 2016, emphasizing ecological restoration as paramount [20]. The Yangtze River Economic Belt spans nine provinces and two municipalities, covering the upper, middle, and lower reaches. This region accounts for 21.4% of China’s territory, contains 40% of its freshwater resources, and sustains 40% of the country’s population and economic output [21]. However, the concentrations of microplastics in the Yangtze River exceed global baselines at levels sufficient to cause ecotoxicological effects [22,23]. In this study, microplastics were detected in all fish samples from the Tongkou River, with counts ranging from one to 25 pellets per fish, corroborating the pervasive microplastic contamination throughout the Yangtze River basin. Every single fish contained microplastics, with contamination levels significantly higher downstream than upstream.

The freshwater fish are widely exposed to small plastic fibers, showing distinct geographic and yearly trends. The finding of this research is consistent with those reported in previous studies [24]. Furthermore, the results indicate that microplastic pollution in the Tongkou River, which serves as the headwater of the Fujiang River—an important tributary of the Yangtze River—has reached a level that warrants urgent attention. This is of considerable significance for the protection of the local aquatic environment and the conservation of aquatic biodiversity.

Trophic strategy and foraging behavior are primary drivers of microplastic (MP) uptake in aquatic organisms. Comparative studies indicate that filter-feeding and herbivorous fish exhibit MP loads that are 3 to 5 times greater than those of omnivorous and carnivorous species, with no significant difference observed between the latter two groups [25]. Although most species examined in this study possess omnivorous diets, their distinct feeding modes and vertical habitat preferences result in critical ecological differentiation. Recent investigations have revealed complex dynamics in vertical MP distribution. Pei et al. documented a higher abundance of MPs in surface-water fish (1.97 items/individual) compared to benthic fish (1.13 items/individual) within the Three Gorges Reservoir, which can be attributed to the accumulation of low-density plastics at the water’s surface [26]. However, this pattern may reverse in the presence of high-density polymers (e.g., PTFE) in bottom waters. Complementary research in Yangtze Estuary aquaculture systems found significantly elevated MP concentrations in crab and shrimp pond sediments compared to fish ponds, likely due to bioturbation-induced fragmentation of plastic films by crustacean burrowing activities [27]. Furthermore, Su et al. reported a 96% MP detection rate in Asian clams (*Corbicula fluminea*) across 21 sites in the middle and lower Yangtze River Basin, underscoring that these typical benthic filter feeders serve as effective bioindicators of aquatic MP contamination [28]. In contrast to the aforementioned studies, all species investigated in this study are typical benthic fish. *H. labeo*, a common benthic omnivore susceptible to microplastic (MP) accumulation, can serve as an effective indicator species for riverine MP pollution. *S. dabryi*, which occupies a stable ecological niche, is a sensitive indicator of habitat contamination. *S. argentatus*, being a dominant population in the Tongkou River, ensures accessibility and representativeness of samples across the river ecosystem. *P. dabryanus*, *T. bleekeri*, *O. dabryi*, and *P. variegatus* exhibit feeding patterns that are closely linked to the sediment environment. Furthermore, their elongated and convoluted intestines prolong food retention time, potentially enhancing MP retention and accumulation within the gut.

China implemented the Plastic Limitation policy in 2007, significantly curtailing plastic bag consumption through mandatory paid usage and the prohibition of ultra-thin plastic bags, thus establishing one of the earliest global benchmarks in plastic pollution governance [29]. In 2017, China further enacted a ban on plastic waste imports, effectively reducing plastic waste leakage at both domestic and international ports while improving national water quality. Nevertheless, this policy inadvertently triggered trade diversion, as several low- and middle-income countries absorbed increased volumes of plastic waste, resulting in a marginal global rise in leakage and underscoring the imperative for coordinated international governance. Notably, the import ban also yielded substantial improvements in river water quality across Guangdong Province, particularly in mitigating heavy metal contamination, by restricting the import and processing of pollution-intensive waste materials. This outcome exemplifies the synergistic interplay between trade policy and environmental governance [30,31]. Hossain et al. applied a decision tree classifier coupled with a Bayesian structural time series model to evaluate microplastic sources and temporal trends in Sansha Bay, China [32]. Their findings revealed that hundreds of tons of microplastics had accumulated in the region over the preceding six decades; however, the overall pollution trajectory exhibited a declining trend. This suggests that the integration of local aquaculture management policies with national plastic restrictions and the foreign waste import ban has been demonstrably effective in reducing microplastic contamination. The present study identifies a statistically significant temporal decline in microplastic pollution, with mean abundance decreasing from 380 pellets per individual in October 2023 to 258 pellets per individual in November 2024, corresponding to a reduction of 32.1% (*p* < 0.05). While this indicates a temporal variation in microplastic loads, these observations are based on a two-year snapshot and may be influenced by seasonal or hydrological differences between the sampling months (October versus November). Therefore, further long-term monitoring is required to establish robust temporal trends. Key measures include: (1) market-driven substitution through the promotion of biodegradable products and the cultivation of green industries; (2) enhanced lifecycle supervision via standardized waste tracking and optimized collection networks; and (3) systemic integration by embedding plastic control within the country’s Zero-Waste City frameworks through dedicated waste management facilities [31]. Despite the foundational role of these national strategic frameworks, the observed interannual variation highlights the necessity of combining macro-level policies with continuous, long-term monitoring. He et al. demonstrated that microplastic concentrations in the Yangtze River system exhibit pronounced longitudinal gradient characteristics, with significantly lower microplastic levels in the upper Jinsha River reaches during the rainy season compared to the middle and lower reaches (*p* < 0.05) [33]. This spatial distribution pattern has been consistently attributed to anthropogenic factors, particularly point source pollution from wastewater discharge and non-point source pollution from agricultural runoff and improper waste disposal. Urbanization intensity shows a positive correlation with microplastic abundance [34]. Wang et al. confirmed that microplastic concentrations in surface waters exhibit a significant negative correlation with distance from urban centers [35]. Similarly, Li et al. reported that oysters collected near urban areas contained higher microplastic loads than those from rural regions [36]. In the present study, the distribution of microplastics in the Tongkou River Basin corroborates these established patterns, while individual-level ingestion showed no significant difference between the upper and lower reaches (Figure 2), the total cumulative abundance of microplastics across sections exhibited an upward trajectory from upstream (149 particles) through midstream (210 particles) to downstream (279 particles). These results indicate that while individual fish maintain comparable baseline ingestion levels, the downstream section bears a heavier collective environmental pressure and higher cumulative input. Supporting the current consensus on microplastic dispersal in freshwater ecosystems. Three key factors govern this process: proximity to urban centers, human activities intensity, and downstream transport dynamics. Notably, these spatial patterns exhibit remarkable consistency across diverse aquatic systems, underscoring the overarching control of anthropogenic factors on microplastic pollution. The findings confirm that urban sources disproportionately impact riverine ecosystems through sustained downstream microplastic fluxes.

Particle size composition analysis is critical for understanding the environmental transport and ecological risks associated with microplastics. The distribution and migration of microplastics in aquatic environments are influenced by a combination of physical, chemical, and hydrodynamic factors. Upon entering water bodies, plastic debris undergoes continuous fragmentation through processes such as photodegradation, mechanical abrasion, and biodegradation [37]. This fragmentation leads to transport behaviors that are significantly affected by particle size and morphology. Our study reveals that microplastics with diameters smaller than 1 mm dominate the samples, comprising 70.38%, while pellets under 2 mm account for 92% across various river sections throughout the year. This size distribution is consistent with findings from Ding et al. in Yangtze tributaries and Qian et al. in bottled water, confirming that smaller microplastics (<1 mm) possess a greater transport potential due to their lower settling velocities, which facilitate long-distance waterborne migration [38,39]. Morphology is another critical factor influencing microplastic transport in aquatic systems. Variations in source, environmental behavior, and biological interactions result in distinct particle shapes. Fibrous microplastics primarily originate from the shedding of textiles, including clothing, ropes, carpets, and the degradation of fishing gear [40]. Fragmented and granular microplastics typically arise from the mechanical breakdown of industrial and consumer plastics, such as packaging and synthetic products [41]. Filmy microplastics mainly derive from agricultural mulch films and plastic sheets. Morphology critically influences the transport and fate of microplastics. Fibers with high aspect ratios and elongated shapes settle more slowly, thereby enhancing suspension and facilitating longer-distance transport [14,42]. This finding aligns with previous studies that have identified fibers as the predominant form in benthic environments, such as sediments from Sishili Bay and aquaculture systems in the Yangtze Estuary [27,43]. Similarly, smaller granular pellets (less than 1 mm) also exhibit strong transport capacity due to their low settling velocities. From a biological interaction perspective, widely dispersed and easily suspended fibrous microplastics are more likely to be ingested and accumulated by benthic fish, which explains the predominant fiber proportion (55.33%) observed among the five microplastic morphologies isolated in this study. The lower proportions of fragments (28.21%), pellets (6.43%), films (8.78%), and rods (1.25%) likely reflect variations in source intensity or environmental fate pathways. This analysis underscores the significance of particle size and morphology in shaping microplastic transport, distribution, and ecological exposure risks within freshwater ecosystems.

Different polymer types of microplastics exhibit distinct toxicological effects on aquatic organisms, largely influenced by their material density, which affects environmental distribution and bioavailability. Low-density polymers, such as polypropylene (PP), commonly used in packaging and water supply pipes, tend to float and are more readily ingested by surface-feeding fish [44,45]. In contrast, denser polymers like polyvinyl chloride (PVC) settle into benthic zones, increasing exposure risks for sediment-associated organisms. However, the polymer composition in aquatic fauna varies regionally due to differences in sources, hydrodynamics, and feeding behaviors. For example, PVC predominated in Hypophthalmichthys molitrix from the Chongqing section of the Yangtze River and Salmo trutta from Mahodand Lake, Pakistan, contrasting with global trends that emphasize the dominance of PP and polyethylene (PE) [46,47]. This study likewise found PVC to be the dominant polymer, consistent with the region-specific variations reported above. The chemical composition of polymers also governs aging patterns and inherent toxicity. Microscopic observations reveal that microplastics undergo surface fragmentation and color degradation, processes driven by hydrodynamic abrasion, photodegradation, and microbial activity [48,49]. Aged microplastics display increased surface area and oxygen-containing functional groups, which enhance their capacity to adsorb co-contaminants such as heavy metals and antibiotics [50]. Aging further elevates hazards associated with PVC by accelerating the leaching of harmful additives like carcinogenic vinyl chloride monomers, potentially impairing biological functions. These material-specific traits promote bioaccumulation through food webs, from plankton to fish and ultimately to humans. Persistent free radicals and reactive oxygen species on aged microplastic surfaces may exacerbate oxidative stress, thereby contributing to chronic health effects. Recent evidence of aged microplastics found in placental tissue highlights their potential to disrupt organ function [51]. Collectively, these findings underscore the intricate interactions among polymer type, aging, and biological impacts within aquatic environments.

These findings collectively demonstrate that microplastic pollution in the Yangtze River Basin poses a significant threat to the aquatic ecosystem. This is evidenced by the widespread accumulation of microplastics in benthic and filter-feeding organisms, followed by their transmission through the food web. Moreover, there is a clear diffusion trend of pollution from urban areas to downstream sections, which directly impacts the health of aquatic organisms and the security of ecosystems. Additionally, specific ecological risks are associated with polymers such as polypropylene (PP) and polyvinyl chloride (PVC) in terms of their transport and toxicity. The following targeted measures are urgently needed: (1) Restoring and constructing riparian buffer zones and vegetation should be prioritized as a natural green infrastructure strategy to effectively intercept and filter land-based plastic debris and runoff before entering aquatic ecosystems. (2) Benthic fish species representing various feeding types (e.g., omnivorous, benthic filter-feeding) and ecological niches should be utilized as effective indicator organisms within biomonitoring frameworks for evaluating microplastic accumulation. (3) It is essential to enhance the removal efficiency of fibrous microplastics in wastewater treatment plants to strictly control urban point-source pollution. (4) Implementing precise source tracing and closed-loop management for textiles and fishing gear, while utilizing green standards and deposit systems, is crucial for achieving source reduction, alongside establishing routine monitoring in downstream urbanized areas and PVC pollution hotspots. (5) There is a need to deepen research on the trophic transfer mechanisms and ecological risks of microplastics, aiming to clarify their transfer patterns from lower to higher trophic levels, including economically important fish species, and the associated health impacts. (6) Furthermore, reinforcing public education to raise environmental awareness, coupled with strengthened regular inspection, monitoring, and administrative supervision by local authorities, is vital to prevent littering and ensure the effective implementation of plastic governance policies.

## 5. Conclusions

Significant interannual and spatial variations in microplastic pollution were observed in the Tongkou River Basin. The overall microplastic abundance in 2023 exceeded that of 2024 by 32.1% (*p* < 0.01), indicating potential fluctuations in pollution inputs or environmental conditions. Spatial analysis revealed pronounced heterogeneity, with downstream sections exhibiting microplastic concentrations 0.78 times higher than those in upstream and midstream areas, reflecting cumulative pollution effects along the river continuum. Fibrous microplastics dominated across all sites, comprising 55.33% of the total, followed by fragments at 28.21%, consistent with global patterns observed in freshwater ecosystems. A strong size-selective distribution emerged, with 70.38% of pellets measuring less than 1 mm. Raman spectroscopy identified three distinct polymer types, including PVC, PP, and cellulose. These findings establish baseline data for microplastic pollution management in the Tongkou River Basin and highlight the ecological risks posed by small-sized fibrous microplastics and PVC contaminants, warranting urgent attention from environmental policymakers and researchers.

## Data Availability

The original contributions presented in this study are included in the article. Further inquiries can be directed to the corresponding author. Data will be made available on request.
